# COVID-19 Pandemic and Periodontal Practice: The Immunological, Clinical, and Economic Points of View

**DOI:** 10.1155/2022/3918980

**Published:** 2022-01-13

**Authors:** Meshkat Naeimi Darestani, Amir Akbari, Siamak Yaghobee, Mina Taheri, Solmaz Akbari

**Affiliations:** ^1^Periodontics Department, Dental Faculty, Tehran University of Medical Sciences, Tehran, Iran; ^2^Finance & Business Economics, DeGroote School of Business, McMaster University, Toronto, Canada; ^3^Dental Implant Research Center, Dentistry Research Institute, Periodontics Department, Dental Faculty, Tehran University of Medical Sciences, Tehran, Iran

## Abstract

The recent global health problem, COVID-19, has had far-reaching impacts on lifestyles. Although many effective WHO-approved vaccines have been produced that have reduced the spread and severity of the disease, it appears to persist in humans for a long time and possibly forever as everyday it turns out to have new mutations. COVID-19 involves the lungs and other organs primarily through cytokine storms, which have been implicated in many other inflammatory disorders, including periodontal diseases. COVID-19 is in a close association with dental and periodontal practice from two respects: first, repeated mandatory lockdowns have reduced patient referrals to dentists and limited the dental and periodontal procedures to emergency treatments, whereas it is important to recognize the oral manifestations of COVID-19 as well as the influence of oral and periodontal disease on the severity of COVID-19. Second, dentistry is one of the high-risk professions in terms of close contact with unmasked individuals, necessitating redefining the principles of infection control. The pressures of the economic recession on patients as well as dentists add to the difficulty of resuming elective dental services. Therefore, this study is divided into two parts corresponding to what mentioned above: the first part examines the clinical and immunological associations between COVID-19 and periodontal and oral diseases, and the second part delineates the measures needed to control the disease transmission in dental clinics as well as the economic impact of the pandemic era on dental services.

## 1. Introduction

Since its outbreak in Wuhan, China, the coronavirus disease of 2019 (COVID-19) turned to a new global concern, taking millions of lives consequently. Although reported with only 2.1% mortality rate (WHO), the disease has placed an intolerable burden in the world as it possesses very high transmissibility and accompanied morbidities. The causative virus, severe acute respiratory syndrome coronavirus 2 (SARS-CoV-2), not only affects the respiratory system but may also lead to a cerebrovascular failure and disorders in other vital organs.

Exaggerated immune responses, e.g., cytokine storm, a common pathway in many inflammatory disorders, are also marked as one of the main tissue damaging mechanisms triggered by COVID-19 [1]. Research suggests that there exists an association between COVID-19 and periodontal diseases as they both show similar proinflammatory cytokine release and affect the systemic health [2]. Accordingly, the present study is aimed at evaluating the mutual relations between the periodontal diseases and COVID-19 and reviewing the immunological, clinical, and practical considerations for dental practitioners.

It also discusses how repeated lockdowns followed by the economic recession have considerably restricted [3] and reduced the demand for dental services including periodontal and dental implant therapies mostly considered as nonemergency treatments.

## 2. The Virus and the Disease Pathophysiology

Coronavirus is a highly contagious common human-animal single-stranded RNA virus, causing mild to severe respiratory infections. In late 2019, a new strand from *β*-coronavirus family, SARS-CoV-2, emerged in Wuhan, China [4, 5] which spread rapidly worldwide, leaving WHO with no choice but to declare the disease as a global pandemic on 11 March, 2020. Previously spread corona viruses (in 2002 and 2012, namely, SARS-CoV and MERS-CoV, respectively) hit a few territories and led to a significantly lower death toll compared to that of SARS-CoV-2. As quantified by the results of PCR tests (on a sample taken from the secretion of the respiratory tract or blood and feces) [4, 6], WHO has reported more than 213 million COVID-19 positive cases and about 4.5 million mortalities (WHO). The unprecedentedly high rate of the disease spread is due to the easy human-to-human transmission through the respiratory aerosols or droplets [7] reinforced by the low mortality rate. Asymptomatic patients, including those spending the 1-14-day latency period, still could be carriers; it is 42% less likely as it is in the symptomatic patients though [8].

The major receptor for SARS-CoV-2 is angiotensin-converting enzyme 2 (ACE2), which binds to the receptor-binding domain (RBD) of the spike protein [9]. The ACE2 receptors are expressed in the respiratory tract and the cardiovascular, central nervous [9], and gastrointestinal systems [4] as well. Following the cell entrance and replication, the SARS-CoV-2 leads to pyroptosis, an inflammatory programmed cell death. In conjunction with the pathogen-associated molecular patterns (PAMPs) and damage-associated molecular patterns (DAMPs), pyroptosis normally stimulates the limited and controlled release of inflammatory cytokines [10]. The excessive immune responses in some patients, however, leads to the cytokine storm and a tissue-wide damage, followed by septic shock and multiorgan (heart, liver, and kidney) damage [10–12]. The disorder finally leading to death is the respiratory failure primarily and also symptoms of sepsis [13]. Severity and complications of COVID-19 are highly associated with dysregulated hyperinflammation in response to viral infection [13].

Several systemic diseases have been found as risk factors for severe COVID-19 and increased mortality rate. Patients with diabetes mellitus are more susceptible to COVID-19 according to the altered immune response. Significantly higher serum level of inflammatory mediators involved in the pathogenesis of COVID-19 is found. The pulmonary viral load may also be elevated during hyperglycemia [14]. Furthermore, the glycemic control of patients with severe COVID-19 is worsened [15].

Higher rate of cardiovascular diseases (CVD), i.e., coronary heart disease and hypertension, has been observed among severe COVID-19 cases and those needing intensive care unit (ICU). This may be due to increased expression of ACE2 in patients with CVD. On the other hand, COVID-19 patients may experience myocardial injury, probably through direct ACE2 receptors, or the cytokine storm induced by altered T-helper response [16]. Obesity, defined as body mass index ≥ 25 kg/m^2^ was also associated with increased COVID-19 severity, characterized by the increased plasma C-reactive protein and lower count of lymphocytes. The supposed predisposing factors in obese patients are the preexisting mild chronic inflammation and reduced innate and adaptive immunity [17].

The exaggerated inflammatory responses, characterized by excessive levels of proinflammatory cytokines (cytokine storm), are common features in COVID-19 and other inflammatory diseases like periodontitis [18]. Periodontitis, a prevalent chronic inflammatory disease, is preliminary initiated by dental plaque but develops and progresses under the influence of the host immune responses [19]. The cytokine storm following the periodontal inflammation releasing into the blood circulation leads to systemic hyperinflammatory responses bound to be amplified by the production of more acute phase proteins (e.g., IL-6 and C-reactive protein) in the liver. This in turn will strengthen the severe inflammation in lungs and other organs. Periodontitis has been associated with several systemic diseases such as diabetes, atherosclerosis, the chronic obstructive pulmonary disease (COPD), and the cerebrovascular disease [20]. Cytokine storm syndrome has been assumed to be the plausible link between COVID-19 and periodontal diseases [1, 21, 22].

## 3. Immunologic Links between COVID-19 and Periodontitis

Below is a list of some common immunological features introduced to explain the possible links between COVID-19 and periodontal diseases.

- *NLRP3 Inflammasome*. Inflammasomes are components of the innate immune system and are formed in response to various pathogen-associated and cellular damage-associated signals. They play important roles in the activation of inflammatory responses and maturation and secretion of proinflammatory cytokines like IL-18 and IL-1*β* [19]. An increasing number of studies have shown the NOD-Like Receptor family Pyrin 3- (NLRP3-) mediated inflammation to be critical in alveolar bone loss and periodontal tissue destruction [23, 24]. In an animal study in 2017, Porphyromonas gingivalis, a keystone periopathogen, accelerated periodontal destruction by NLRP3 inflammasome activation which was followed by a response from the IL-1 family, while significantly less proinflammatory responses were observed in NLRP3-deficient mice [25]. NLRP3-stimulation has been shown to intensely induce cytokine expression during the cytokine storm in COVID-19 and periodontitis [20]. SARS-CoV-2 might directly activate NLRP3 inflammasome, and dysregulated NLRP3 inflammasome activity has been attributed with severe forms of COVID-19 with tissue damage and relevant cytokine storm [26, 27]. Potential therapies targeting inflammasomes have been recently introduced for the treatment of COVID-19 and periodontitis. Melatonin is a well-known immunomodulator with anti-inflammatory and antioxidant properties, preventing the activation of NLRP3 during inflammation. Several studies support the beneficial properties of Melatonin in the treatment of COVID-19 [28, 29]. Melatonin has also been demonstrated to prevent periodontal destruction in experimental periodontitis in rats [30].
- *Interleukin-17 and T-helper 17-Mediated Responses*. Patients with severe forms of COVID-19 and periodontitis have been reported to suffer from elevated serum levels of several cytokines (like IL-1beta, IL-8, IL-9, IL-10, IL-17, G-CSF, IFN-*γ*, and TNF-*α*). Many of these cytokines have critical parts in stimulating TH17-type innate immune responses. The T-helper 17-mediated responses are supposed to be involved in the cytokine storm and resultant tissue damages made by a variety of pulmonary viral infections including H1N1 influenza and SARS-CoV-2 [31]. Interleukin-17 also plays a pivotal role in pathogenesis of periodontitis [32]. Increased crevicular and salivary levels of IL-17 have been reported in chronic/and aggressive periodontitis as compared with healthy subjects [33, 34]. Several studies have demonstrated the efficacy of cytokine inhibitors (Biologics) that manipulate IL-17 pathways in the management of several immune-mediated inflammatory diseases. Such therapeutic agents are proposed as promising strategies to prevent the deteriorating outcomes of TH17-associated cytokine storm in COVID-19 and periodontitis [32].
- *Neutrophil Extracellular Trap (NET)*. There is growing evidence to support the role of neutrophil-induced immunopathogies in the development of periodontitis and the severe forms of COVID-19. Neutrophil extracellular trap (NET) release is one of main actions of neutrophils and functions through mechanical entrapments of bacteria, viruses, and any other insults. But in several clinical conditions, NET generation (NETosis) leads to the host's cellular damage and death, either directly or through autoimmune mechanisms (working as a double-edged sword) [35]. To date, compelling evidence demonstrates the correlation between severity of COVID-19 and neutrophil count as well as activation [35]. NETosis can be directly triggered by viable SARS-CoV-2 from healthy neutrophils [36]. In a case-control study on 16 hospitalized COVID patients, blood samples were obtained for 11 consequential days. Circulating neutrophils exhibited exaggerated NETosis and oxidative burst compared to the figures in the control group [37]. NETosis and relevant mediators such as interferon-alpha have been also found to play a potential role in the pathogenesis of periodontal diseases [38, 39]. Owing to the enhanced levels of NETs in both COVID-19 and periodontitis and its implication in cytokine storm, patients suffering from periodontitis may be at a higher risk for developing severe types of COVID-19 with more complications [40].
- *Interleukin-*6. Several immune cells (like macrophages and CD4+ T-cells) and nonimmune cells (fibroblasts and endothelial cells) produced IL-6. Interleukin-6 can induce T-helper1 and T-helper 17 cytokines, activate B-lymphocytes, and influence myeloid cell differentiation. IL-6 families play especially important roles in the pathogenesis of periodontal disease and many other inflammatory diseases and are correlated with clinical severity of periodontal diseases [41]. The important roles of IL-6 in the pathogenesis of cytokine storm have already been elucidated, but recently, its functions in the pathogenesis of COVID-19 have received more attention [42]. Several studies reported that SARS-Cov-2 infection selectively induced IL-6 production, and in some cases, IL-6 level increased up to 1000-fold above normal ranges. This marked elevation of IL-6 level can result in lymphopenia, immunoparalysis, and adverse clinical outcomes [43]. Two meta-analyses on complicated COVID-19 cases detected higher levels of IL-6 (2.9-fold) compared with those with nonsevere conditions [44, 45]. IL-6 inhibitors have been widely used as an effective therapeutic modality in the treatment of several immune-mediated inflammatory diseases, and recently, promising results have also been achieved with some anti-IL-6R monoclonal antibodies like Tocilizumab [42]. IL-6 has been found to have similar countless functions in the pathogenesis of the cytokine storm associated with both periodontitis and COVID-19, supporting the possible links between these two diseases.

## 4. COVID-19 in Periodontal Patients

A number of case-control studies have evaluated the correlation between periodontitis and the risk/severity of COVID-19. In a study on 568 COVID-19 patients, periodontitis has been found to be associated with higher risks of severe complications of SARS-CoV-2 virus infection (death, ICU admission, and the need for assisted ventilation). Here, forty subjects suffered from severe complications (cases), and 528 patients were discharged without any complications (controls). The presence and the severity of periodontal disease were diagnosed based on radiographic data. Since the relation between the periodontal disease and COVID-19 may be due to the common risk factors [46], potential confounders such as sex, age, body mass index, smoking, any chronic respiratory disease, heart diseases, diabetes, and autoimmune diseases/or immunosuppressive conditions were adjusted. The risk of advanced COVID complications was found to be higher in patients with moderate to severe periodontal disease than in either the healthy ones or patients with mild periodontitis [47]. In another case-control study, periodontal status and oral hygiene conditions of 79 patients with positive COVID-19 PCR results were compared with 71 control subjects (negative PCR results). Significant associations were found between COVID-19 and plaque scores ≥ 1 (OR, 7.01), gingivitis (OR, 17.65), and severe periodontitis (OR, 11.75) [48]. In a study by Gupta et al., only 5 out of 13 symptomatic COVID-19 patients had systemic comorbidities, while 6 patients had periodontal diseases [49]. Findings also showed the relationship between the dental damage stage (radiographic bone loss and caries) and the morbidity and mortality rate of COVID-19. In other words, the higher was the dental damage stage, the greater was the hospitalization rate, probably indicating a relationship between dental health status and the severity of COVID-19 [50].

## 5. Oral Manifestations of COVID-19

### 5.1. Oral Lesions

Similar to several viral infections, COVID-19 may arise various forms of oral lesions in both keratinized and nonkeratinized mucosa [51]. The onset of oral lesions usually coincides with the taste and smell chemosensory dysfunctions, and severity of oral lesions is mainly influenced by an older age and severity of COVID-19 [52]. Oral manifestations of COVID-19 include a wide range of presentations including sores, erosion, vesicles, bulls, pustules, depapillated tongue, macules, papules, plaque, mucosal pigmentation, halitosis, whitish areas, necrosis, petechia, swelling, erythema, and internal bleeding. The most and least common involved sites were tongue and tonsils, respectively. Gingiva has been reported to be involved in less than 10% of cases. The oral lesions were often symptomatic (painful, burning, or itchy), and there were no sex differences [52]. The oral lesions mostly appeared about 4 to 7 days after the onset of systemic symptoms; earlier or later (up to 12 weeks) onsets have been also reported though. The lesions persisted only from 3 to 28 days after appearance [53]. Without age limitations, lesions could be detected even in children [54]. The etiology of oral lesions is still unclear, but it seems that the COVID-19 infection is not the primary cause as mucosal lesions with both a nonspecific pattern and various forms associated with a particular virus are rare [55]. The oral lesions have been attributed to various factors: bacterial/or viral coinfection, hyperinflammatory response secondary to COVID-19, adverse drug reactions, and patients' deterioration of the general health state. The Aphthous-like lesion is a common form of oral lesions in COVID infection. It is mostly seen on the borders of tongue, inner side of lips, and buccal mucosa, appearing as a shallow ulcer with erythematous borders and yellow-white pseudomembranes. Elevated levels of tumor necrosis factor- (TNF-) *α* in COVID-19 patients have been attributed with the development of Aphthous-like lesions [52]. Secondary herpetic gingivostomatitis has also been reported in the context of COVID-19 infection [54, 56]. The lesions manifest as multiple small circumscribed ulcerations of the oral mucosa, mostly detected on keratinized mucosa (gingiva and palate), and covered by yellow–gray membranes. Oral candidiasis is another oral lesion in COVID-19 patients. In a case report study, an asymptomatic COVID-19 patient has been diagnosed just by oral candidiasis lesions in the form of petechiae on the lower lip and melanin pigmentation in the gingiva [57]. The long-term use of broad-spectrum antibiotics, prolonged inpatient care, poor general health, and oral hygiene following COVID-19 infection were causal factors of immunosuppression, leading to these manifestations. The oral lesions related to COVID-19 infection have received several treatments, including chlorhexidine mouthwash, local/or systemic antifungal therapy (nystatin and oral fluconazole), systemic or topical corticosteroids, systemic antibiotics, systemic Acyclovir/Penciclovir, artificial saliva, and photobiomodulation therapy. The factors mostly involved with the oral lesions in COVID-19 patients could be outlined as poor oral hygiene, opportunistic infections, stress, underlying diseases, trauma (following intubation), vascular compromise, and hyperinflammation response following virus infection [53].

### 5.2. Taste Dysfunction

Taste dysfunction (dysgeusia) and loss of smell (anosmia) are common features in COVID-19 patients and can be the initial and the only manifestations as well [58]. In a recent systematic review on the patients with COVID-19, the prevalence of olfactory and gustatory dysfunction was reported to be about 52% and 25-60%, respectively [59]. SARS-CoV-2 virus infection could affect gustatory function in several possible ways: (i) ACE2 receptors, as the main receptors of SARS-CoV-2 virus, were spotted on the epithelium and also in the ductal elements of minor salivary glands [60]. The viral infection of such cells may result in either quantitative or qualitative changes in saliva secretion and subsequently taste dysfunction [61]; (ii) there are robust links between the gustatory and olfactory functions. Accordingly, another source of taste disturbance could be anosmia, a disorder that is triggered by the damage SARS-CoV-2 virus invasion makes to the olfactory epithelium; (iii) direct impairment of neurosensory pathways of gustation may also account for taste dysfunction. The symptom might be developed by viral colonization and resultant damage to ACE2-expressing cells of the taste buds [62] and peripheral taste neurosensory chemoreceptors as well as damage to any relevant cranial nerves [59, 63–65]. Taste disorders are likely to be significant and highly specific symptoms in patients with mild to moderate COVID-19 and are known as important markers in primary infection. Therefore, any patients presenting with a smell and taste disorders are recommended to undergo further evaluations for COVID-19 infection [65].

### 5.3. Xerostomia

The other common oral manifestation in patients with COVID-19 is xerostomia [66]. The presence of ACE2 in salivary gland tissues and invasion of SARS-CoV-2 via ACE2 into these cells provide a possible explanation for quantitative and qualitative salivary disorders in COVID-19 patients. Xerostomia has been reported in 32-50% of COVID-19 patients and could occur as one of the first symptoms of the disease even before COVID-19 diagnosis [67, 68]. In a case-series study on 10 COVID-19 patients, 60% of cases experienced xerostomia a few days before any other symptoms and simultaneously with or 1–2 days after the onset of other symptoms [69]. It is noteworthy that the evidence regarding the pathophysiological mechanism how SARS-CoV-2 infection causes the dry mouth and taste disorders is limited to studies with low sample size and limited hypotheses [58]. Therefore, more studies are needed to unveil the underlying causes.

## 6. The Significance of Oral Hygiene in COVID-19

The shift in the composition of dental biofilm, from health-related microorganisms to the disease-associated ones, i.e., dysbiosis, may lead to an increase in the proportion of periodontopathogens resulting in periodontal diseases. The oral dysbiosis creates a suitable environment to retain the respiratory pathogens [46]. COVID-19 patients are more prone to oral dysbiosis due to impaired immune response, medications, and dietary changes. In addition, in critically ill patients due to weakness and hospitalization, oral hygiene is impaired, which exacerbates dysbiosis [70–72]. Several systematic reviews indicated a positive association between poor oral hygiene and oral care interventions in nursing home and hospital patients [73–75].

In hospitalized COVID-19 patients, the incidences of secondary pulmonary infections were reported 16% for bacterial infections and 6.3% for fungal infections [76]. Zhou and colleagues showed that half of the patients who expired due to COVID-19 experienced a secondary infection and ventilator-associated pneumonia (VAP), occurring in one-third of patients in need of mechanical ventilation [77]. Several bacterial species from oral microflora (including main periopathogens) may rapidly migrate from mouth and upper airways to the lower respiratory tract especially during the orotracheal intubation and develop respiratory diseases or contribute to the pathogenesis of ventilator-associated pneumonia [78]. Zijie and colleagues isolated oral bacteria from the bronchoalveolar lavage fluid taken from the patients with COVID-19 [79]. An in vitro study observed the upregulation of ACE2 receptors on human respiratory epithelial cells after they were exposed to Fusobacterium nucleatum (one of the main red complex periopathogens) [80]. A growth in the concentration of ACE2 receptors increases the viral binding potential and infectivity. Furthermore, proteases derived from periopathogens were found to activate virus Spike protein [81] and enhance virus pathogenicity [49].

On the other hand, several systemic diseases or conditions affecting the immune system may initiate the oral dysbiosis. Viral infections, e.g., influenza and SARS-CoV-2, are shown to be associated with alterations in the oral flora [82]. Common inflammatory cytokines, such as increased IL-17 levels, are found between periodontitis and COVID-19 patients [21].

Several studies have reported that improved oral hygiene, whether by mechanical or chemical plaque control measures, can reduce oral bacterial burden giving rise to respiratory infections and consequently reduce the risk of mortality (by about 60%) following the aspiration of the pathogens [83, 84]. Brushing the teeth and cleaning the tongue at least twice a day and oral rinsing with antiseptic solutions (like hydrogen peroxide 0.5-1.5%, cetylpyridinium chloride 0.1-1.5% or povidone iodine 0.23-1% [85], 1.5% hydrogen peroxide, mixture of cetylpyridinium chloride, and povidone iodine) all effectively reduce oral bacterial load [86–88]. Studies have shown that these products effectively inactivate SARS, MERS, and H1N1 in 1 minute [87–90]. Finally, oral hygiene should be well improved, especially in ICU and the patients over 70, if anyone seeks to reduce risk of aspiration pneumonia and COVID complications [66].

## 7. Protection against COVID-19 Pandemic Using Periodontal Treatments

Treatment of oral and dental diseases and removal of the bacterial biofilm also contribute to preventing severe types of COVID-19. However, dental treatments with the production and distribution of aerosols raise the concern of disease transmission from patients to oral healthcare providers [91].

Although vaccination with different types of WHO-approved vaccines or other types has been in practice across the globe, it should be noted that their generated immunity is lower against newer types of coronavirus mutations. Variants of concern (VOCs) alpha, beta, and gamma are sensitive to neutralization in the serum of convalescent or vaccinated individuals, albeit with less sensitivity [92]. The Delta variant, however, is about 3 to 5 times less likely to be neutralized by the serum antibody in people who have received two doses of Pfizer or Astrazeneca vaccines [93]. Therefore, precautions are still very important to prevent the spread of the disease.

Screening the patients for symptoms, such as fever, is a primary preventative measure (CDC guideline) before any dental treatments. In vitro studies have shown the ability of oral antiseptic mouthwashes to inactivate viruses, so chlorhexidine [94], hydrogen peroxide, and povidone iodine [95] rinses are probably beneficial prior to aerosol-generating procedures (AGPs). Also, topical antimicrobial solutions could be administered by nasal spraying or douching. Rinsing the mouth and nose can reduce effectively the exposed viral load, which is beneficial to prevent the disease or reduce its severity [96]. Carrageenan, a carbohydrate found in red seaweed, has been tested as an antiviral agent in nasal sprays, and following their application, nasal viral load virus decreased with no clinical improvement [97–100]. The use of highly effective respirators, including FFP2, FFP3, and/or N95, as well as the use of protective shields significantly reduce the risk of transmission of viruses to the dentist's mouth, nose, and eyes [101]. In order to minimize the risk of SARS-CoV-2 cross infection between virus carrier and oral healthcare providers or other patients, there exist other important measures including the use of high-volume evacuator to reduce the spread distance of droplets and aerosols [102], proper space ventilation, and sufficient time interval and distance between the patients. A recent study illustrated the positive effect of adding high molecular weight FDA-approved polymers to dental unit irrigation solutions to reduce aerosol production during scaling with ultrasonic devices [103]. Furthermore, using manual scaling and root planning instruments instead of ultrasonic devices is an alternative nonaerosol generating procedure, although it is more time-consuming [86]. The droplets made after ultrasonic scaling are able to travel long distances. Therefore, as the sensitivity of SARS-CoV-2 dictates, any related surface must be very well disinfected in compliance with the usual protocol for surface disinfection and sterilization of devices [87].

## 8. Economic Implications

Restrictions on dental services have also created financial problems for dentists and oral health professionals. These can be envisioned in three channels: the supply of dental services, the patients' demand for these services, and the increased cost of operations.

The supply of dental services is directly restricted by the local governments and policymakers. Most dental procedures provided during the lockdown period have been limited to emergency needs [88], and elective treatments have been prohibited according to ADA guidelines [89]. Moreover, COVID-19 guidelines warrant fewer patients to be present in dental clinics, and more stringent, time-consuming, cleaning procedures are to be observed after each patient has left the clinic. These shrunk the capacity of dental clinics to offer services for elective treatments in particular. In the meantime, the pandemic has resulted in an economy-wide recession and unemployment, hitting hard the budget constraints of most individuals in the economy. Research has documented that during recessions, for instance, the one in 2008 in the United States or the one in 2012 in Greece, consumers cut nonessential expenditures and increase their precautionary savings to cope with the unknown consequences of the possible long economic meltdown [90]. The income loss, together with the change in the consumption behavior, reduced the affordability of the nonessential needs and more specifically of the more expensive dental treatment such as implant treatments [104]. The third channel through which COVID-19 has financially affected dentistry is the increased cost of operations. The disruptions in the global supply chain have increased the price and scarcity of dental service supplies. In addition to these, providing extra personal protective equipment (PEE) and implementing further cleaning protocols to control the spread of COVID-19 have imposed additional costs to clinics. In private conversations with dentists, we arrived at the conclusion that many were forced to delay treatments and operations for their patients due to the unavailability of supplies and materials. In total, these reinforcing channels negatively affected the current and future sources of income for dentists and their staff.

Survey data and simulation exercises also suggest that COVID-19 brings about negative financial consequences for this sector. For instance, Schwendicke et al. [105] used a scenario analysis on German healthcare providers and found that the revenue from the public insurance or private insurers or out-of-pocket expenses had reduced on average by more than 18.7%. In an Iranian dentists' response report, 97% of surveyed dentists said that their income had decreased during the pandemic, irrespective of the dentist's specialty [106]. A survey conducted in Brazil showed that 9 out of 10 dentists were worried about the economic impact of quarantine [88]. It is even argued that the decrease in demand, followed by a decrease in the income of dentists, reduces the number of private practitioners who may not be able to restart after the pandemic [107]. The closure of dental clinics results in the unemployment of many office workers [88]. These may indicate the need for governmental support for dentists and dental clinics.

For the policymakers, it is important to disentangle the hesitations of dental service providers to offer such services from their inability to do so. If some healthcare providers choose not to work during the peaks of the pandemic, a different incentive package is required to simulate the sector compared to the scenario that dental clinics are not able to offer their services due to public health guidelines. Similarly, it is necessary to more clearly identify the patients' demands in response to COVID-19 shock. Firstly, the classification of dental services to elective and emergency procedures is not objective but is patient-dependent. For instance, a patient in the middle of an implant procedure might perceive the final steps of the treatment as an essential service, whereas a patient who has not initiated the implant procedure might evaluate it elective during the lockdown periods. Second, the COVID-19 shock has imposed an asymmetrical income loss for the individuals in the economy. It is reported that the more skilled workers able to work remotely have even experienced a positive income shock, possibly due to the increased efficiency. In sum, these confounding forces convolute the identification of the effect of COVID-19 on the supply and demand of dental services.

## 9. Conclusion

According to the very limited evidence mainly of low quality, it is supposed that the periodontal disease could be a risk indicator of the COVID-19 severity. Early detection and treatment of periodontal disease, as well as identification of hyperresponsive individuals through cytokine profiling, may assist in selection of appropriate anticytokine drugs. Though promising outcomes have been reported following treatment of patients with COVID-19 with immunomodulators (e.g., IL-6 inhibitors), further clinical trials are required to understand the efficacy and safety of these drugs according to disease stage and severity. On the other hand, complicated COVID-19 patients are more susceptible to oral dysbiosis due to impaired immune response as well as inability to maintain oral hygiene, which exacerbates periodontal health. So improved oral hygiene is highly recommended especially in hospitalized patients. Dental procedures encountered obvious drawbacks due to the financial recession and high risk for transmission of the disease through the measures. In spite of vaccination and increased knowledge about the disease, the new variants are still addressed with precautions; their long-term complications are not yet evaluated. Equally, fresh measures must be taken to tackle the probable future pandemics.

## Data Availability

The datasets used and/or analyzed during the current study are available from the corresponding author on reasonable request.
